# The Russian Bioimpedance Database: An Update

**DOI:** 10.2478/joeb-2022-0010

**Published:** 2022-11-23

**Authors:** Sergey G. Rudnev, Olga A. Starunova, Elena Z. Godina, Alla E. Ivanova, Alexander V. Zubko, Vladimir I. Starodubov

**Affiliations:** 1 Marchuk Institute of Numerical Mathematics, Russian Academy of Sciences, Moscow, Russia; 2 Federal Research Institute for Health Organization and Informatics, Ministry of Health of the Russian Federation, Moscow, Russia; 3 Anuchin Research Institute and Museum of Anthropology, Lomonosov Moscow State University, Moscow, Russia

**Keywords:** Bioimpedance data, large database, phase angle

## Abstract

Extensive bioelectrical impedance analysis (BIA) data have the potential of health monitoring and the assessment of health risks at the population level. The importance of BIA data lies in their availability and abundance for many countries. In Russia, mass BIA data are generated by the national network of health centers (HCs). Our aim was to describe the structure and capabilities of the updated HCs’ BIA database. Upon several requests between 2012 and 2020, 369 HCs representing all Federal districts of Russia and 60 out of 85 Federal subjects in them, submitted raw bioimpedance data which were obtained using the same type of BIA instrument, namely ABC-01 ‘Medas’ (SRC Medas, Russia). After application of strict selection criteria, 2,429,977 BIA measurement records were selected that formed the updated 2010-2019 HCs’ database. Various slices of the BIA data are described according to spatiotemporal, demographic and other characteristics. Reference curves of the bioimpedance phase angle according to age and sex are presented. Limitations and prospects for further work are outlined. We believe that, after appropriate sampling, the database can be utilized to study biological, geographical, social and other associations of the bioimpedance and body composition parameters, for generating updated national references, international comparisons and data standardization.

## Introduction

Due to ease of use, noninvasiveness, portability, reliability and relatively low cost, bioelectrical impedance analysis (BIA) is now one of commonly used body composition assessment methods with numerous applications in medicine and biology [1, 2, 3]. At present, big databases of BIA measurements are formed and utilized, among other things, for generating national reference data and the assessment of population health [4, 5, 6, 7]. In particular cases, manufacturers report an ongoing global BIA data collection process through the network and mobile technologies based on their instruments with the implementation of a big data approach using huge multi-million datasets of BIA records [8]. On the other hand, there are examples of meta-analysis of massive aggregated BIA data from different manufacturers [9].

**Fig.1:**
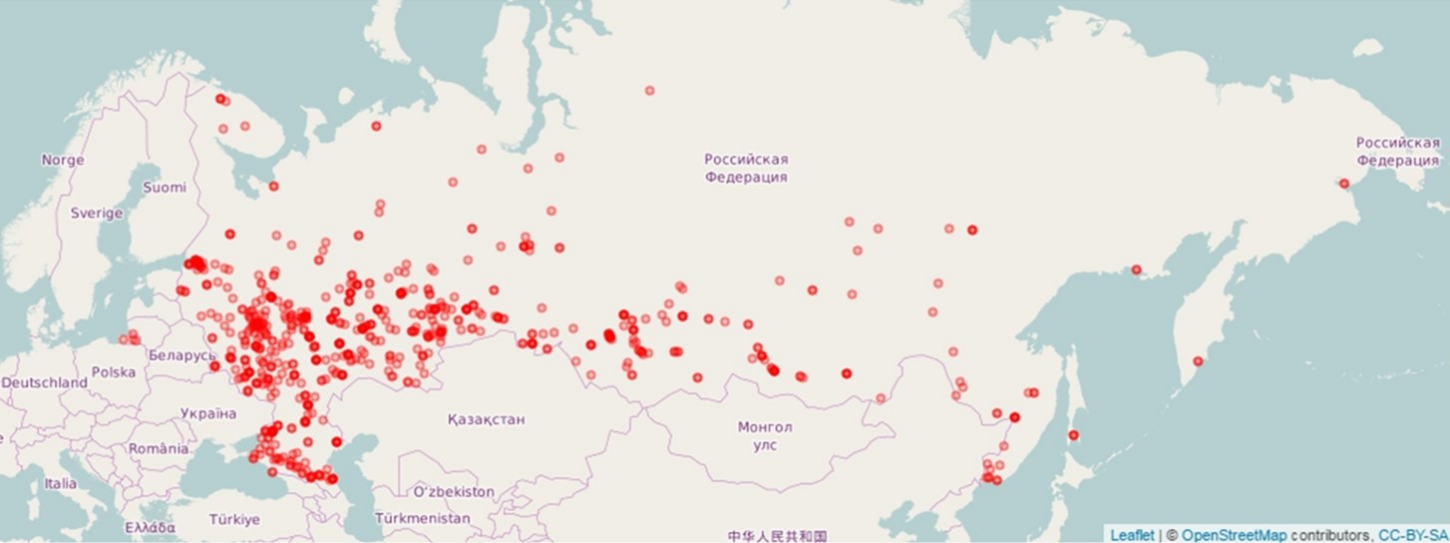
The Russian health centers – geographical distribution.

In Russia, mass BIA data are generated by the national network of health centers (HCs) which was established in 2009-2010 for the assessment of individual health and promotion of healthy lifestyle. Currently, this network involves nearly 800 HCs evenly distributed according to the population density at the approximate rate of 1 HC per 200 thousand people (figure 1). Once a year, on a free voluntary basis, every citizen of Russia has the opportunity to pass a comprehensive examination at any HC with the use of several assessment methods, including measurement of height, weight, blood pressure, cholesterol, glucose, blood oxygen saturation level, the assessment of ankle brachial index, waist-to-hip ratio, eye examination, smoking test, spirometry, wrist dynamometry, ECG dispersion mapping and, optionally, BIA. As a result of the examination, each patient is given current recommendations by a nutritionist on maintaining a healthy lifestyle.

The 2010-2015 HCs’ BIA database was used previously for the construction of the population reference data, analysis of associated health risks and inter-population comparisons [10] as well as to develop the data filtration algorithms and related software [11]. The new data collection was then organized in 2017 and 2019.

Our aim was to describe the structure and capabilities of the updated HCs’ BIA database.

## Materials and methods

Bioimpedance was measured in HCs during visits between 2009 and 2020. BIA data were collected from HCs in a depersonalized form in several stages. The data for 2018-2019 were obtained according to the Letters of the Federal Research Institute of Health Organization and Informatics no 7-5/1498 of 17 Nov 2019 and no 7-5/1020 of 31 Aug 2020. The 2015-2017 data were obtained according to the Letter no 7-5/1067 of 27 Nov 2017. The data for the previous years were obtained according to the Letter of the Ministry of Health of the Russian Federation no 14-1/10/2-3200 of 24 Oct 2012 as well as from the Federal Information Resource of HCs as described in [10] and also according to the Letter no 7-5/434 of 02 Jul 2015. Only BIA data from the ABC-01 ‘Medas’ instrument (SRC Medas, Russia) were considered which were submitted by 369 HCs. The data for 2009 and 2020 were rare and so neglected.

The bioimpedance instrument АВС-01 ‘Medas’ (SRC Medas, Russia), with the Russian abbreviation ‘ABC’ meaning ‘analyzer of water compartments’, represents a family of phase-sensitive two-, six-, or multiple-frequency lead-type BIA meters generating a low-intensity (800 μA) electrical current and utilizing mainly tetrapolar or, optionally, octopolar electrode configuration. Of them, two-frequency tetrapolar meters are used in HCs. Currently, these devices have been discontinued, and ABC-02 ‘Medas’ instruments are being manufactured instead. With these instruments, body composition is evaluated using known published equations, such as for fat-free mass (FFM) in children [12] and total body water in adults [13].

For every patient attending HC either individually (in case of adults) or with parents or as part of organized school team (in case of children), the inclusion criterion for the BIA was age 5 or older, and the general exclusion criteria were pregnancy or the presence of a cardiac stent or pacemaker.

BIA measurements were carried out after a few minutes of patient’s staying indoors, with minimal clothing, on the right side of the body in the lying position with arms and legs slightly abducted according to a conventional tetrapolar scheme at the electrical current frequencies 5 and 50 kHz with the use of disposable bioadhesive electrodes. For every HC, information about the type of the electrodes and the mode of BIA application – either selectively (e.g., in case of overweight) or to the entire flow of patients, was collected. F9049 (FIAB, Italy) and Schiller biotabs 22×34 mm (Schiller, Switzerland) ECG electrodes were utilized in HCs most often accounting for about 90% of the measurements followed by Top Trace Medtab (Ceracarta, Italy), Skintact RT-34 (Leonhard Lang GmbH, Austria), Eurotrode PFR2034 (Pirrone srl, Italy) and other types of electrodes. Phase angle (PA) was calculated at 50 kHz as PA=atan(Xc/R)×180°/π, where Xc is the reactance, and R the resistance.

Standing height and weight were measured in HCs using mainly the same type of electronic stadiometer and digital scale manufactured by JSC Tulinovsky instrument making plant ‘Tves’ (Tambov region, Russia) with an accuracy 0.5 cm and 0.1 kg, respectively.

As such, the BIA instrument ABC-01 ‘Medas’ provides stable measurements with a relative error value of about 0.1% for R and 1% for Xc. The quality control of BIA measurements in HCs using the ABC-01 ‘Medas’ instrument was provided through the initial instruction of the personnel on working with the analyzer from the manufacturer as well as due to organized annual training sessions of the HCs’ staff in Moscow. In addition, to check the performance of the BIA instrument itself, it was recommended in HCs to daily measure the supplied equivalent electrical circuit before starting measurements of patients making sure that the differences between measured and proper values of the resistance and the reactance, respectively, are minimal.

A joint study conducted in the fall of 2020 at Medical Computer Systems company (Zelenograd, Russia) included 9 replicate BIA measurements of 20 participants (10 women and 10 men, age 21-54 years, BMI range 18.5-32.0 kg/m^2^) with the ABC-01 ‘Medas’ instrument using 8 different types of disposable bioadhesive electrodes: Ambu White Sensor 0415M (Ambu, Denmark), Bianostic AT (Data Input GmbH, Germany), Eurotrode PFR2034 (Pirrone srl, Italy), F9049 (FIAB, Italy), Schiller biotabs 22×34 mm (Schiller, Switzerland), Skintact RT-34 (Leonhard Lang GmbH, Austria), Top Trace MedTab (Ceracarta, Italy), and Vermed (Nissha Medical Technologies, Japan). All of them, except for the Bianostic AT electrodes specially developed for BIA, were reported to be utilized in HCs. The electrodes were applied in random order, with the first and the last patient’s measurement using the same electrode type. The interelectrode technical error of measurements TEM=$\sqrt{\sum_{i=1}^{N}\left(\sum_{j=1}^{K}M_{ij}^{2}-{\left(\sum_{j=1}^{K}M_{ij}\right)}^{2}/K\right)/N(K-1)},$where *M_ij_* is the result of *j*-th measurement for *i*-th participant, *N* – the number of participants (*N*=20), and *K* – the number of electrode types (*K*=8) [14], was 3.86 ohm R, 0.52 ohm Xc, 0.07° PA, 0.26 kg FFM, and 0.33% percentage body fat (data not published). Interestingly, for the most frequently utilized in HCs electrodes F9049 (FIAB, Italy) and Schiller biotabs 22×34 mm (Schiller, Switzerland), we observed the minimal inter-electrode differences in BIA parameters of 1.30 ohm R, 0.26 ohm Xc, 0.02° PA, and 0.04% percentage body fat.

In a limited joint study conducted in the spring of 2018 at the Anuchin Research Institute and Museum of Anthropology of MSU (Moscow, Russia) with replicate measurements of 5 participants (4 women and 1 man, age 25-45 years, BMI range 20.1-24.9 kg/m^2^) using 4 different ABC-01 ‘Medas’ instruments and F9049 (FIAB, Italy) electrodes, the inter-instrument TEM, which was calculated using the above formula, where *M_ij_* is the result of measurement of *i*-th participant by *j*-th instrument, *N* – the number of participants (*N*=5), and *K* – the number of BIA instruments (*K*=4), was 2.01 ohm R, 1.19 ohm Xc, 0.10° PA, 0.07 kg FFM, and 0.12% percentage body fat (data not published).

**Fig.2:**
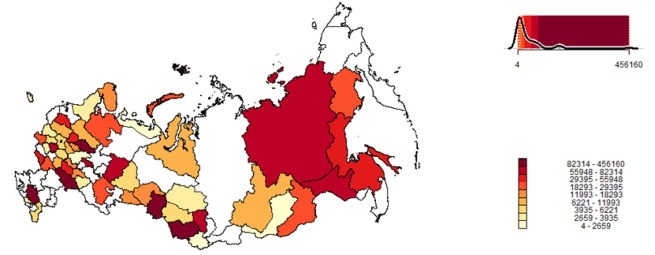
Frequency distribution of the BIA records (n=2,429,977) by the Federal subjects of Russia.

The BIA data collected at various stages had common measurement records. For the removal of duplicates, a quasi-unique identifier was used for each record based on the HC’s name, date of birth, date of measurement and patient’s sex. In addition, all but the last repeated measurement records during the same visit were excluded. BIA records were also excluded if they did not contain information on height, weight, electrical resistance and reactance, date of birth, date or time of measurement, and patient's sex. The obtained initial dataset of BIA measurements for 2010-2019 contained 4,162,925 records. Strict selection criteria were then applied in order to remove outliers and fraud data according to the developed expert quality assessment algorithm [11], and the database of 2,429,977 selected measurement records was formed. Distribution of the selected BIA data across the Federal subjects of Russia is shown in figure 2.

The structure of the total number of examined patients was described relative to age, sex, time of examination and year of birth. Age-related changes of the phase angle in males and females were assessed. Statistical analysis was performed using the Minitab 21 and MS Excel 2019 software packages.

## Informed consent

Informed consent to the collection, processing and use of personal data has been obtained in HCs from all individuals included in this study, or their legal representatives – for children under 14 years of age.

## Ethical approval

The research related to human use has been complied with all relevant national regulations, institutional policies and in accordance with the tenets of the Helsinki Declaration. Due to the use of routine diagnostic methods and the lack of medical interventions, the permission of the ethics committee was not required.

## Results

**Fig.3:**
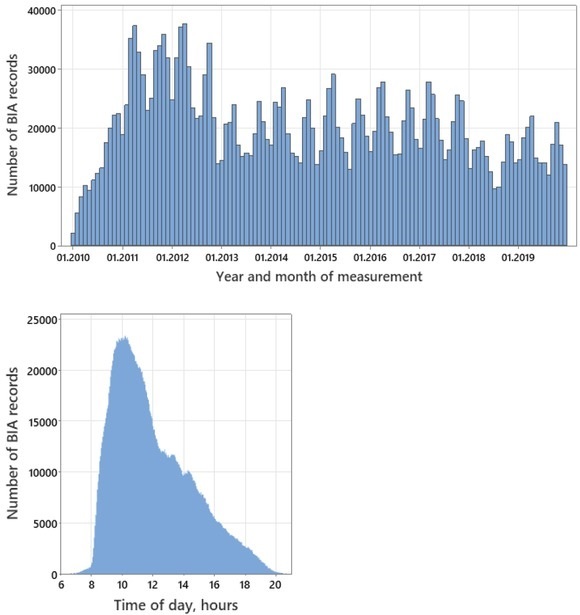
Frequency distributions of the BIA records (n=2,429,977) by the month and year of measurement (upper panel) and the time of day (lower panel).

For the observed time period, the monthly number of BIA records in the database ranged basically from 10 to 30 thousand and showed a pronounced seasonality with the local maxima in the spring and autumn seasons, and the local minima in the winter and summer seasons, respectively (figure 3, upper panel). The maximal rate of visits occurred in the morning hours, from about 9 am to 12 am (figure 3, lower panel). The maximal rate of visits on workdays was observed, on average, on Wednesday with a decrease in the intensity of visits towards the beginning and the end of the week, while the range of differences in the frequency on different working days of the week did not exceed 10-12% (data not shown).

**Fig.4:**
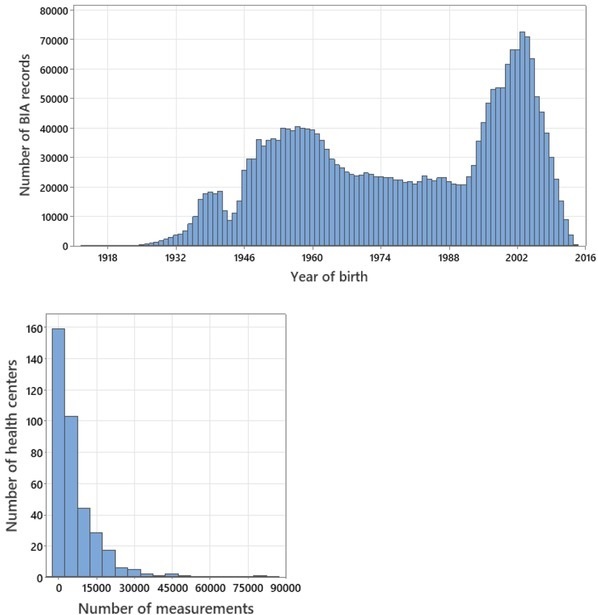
Frequency distribution of the BIA records (n=2,429,977) by the year of birth of the measured persons (upper panel) and of the total number of HCs according to the number of BIA measurements (lower panel).

The children born in 1997-2007 were examined in HCs most frequently (Fig. 4, upper panel) followed by those people who were born in 1949-1962. People who were born in 1942-1945 were examined relatively rarely, which represents the demographic echo of WW2. The distribution of the total number of HCs according to the number of measurements (Fig. 4, lower panel) reflects the fact that at least some HCs carried out BIA measurements selectively, and not to the entire flow of patients. In addition, HCs did not always provide data in response to every request (or could even be closed or reorganized), so the entire data set of each individual HC was available quite rarely. The median number of BIA records in the database per one HC was 3260, with the interquartile range between 908 and 9050 records.

**Fig.5:**
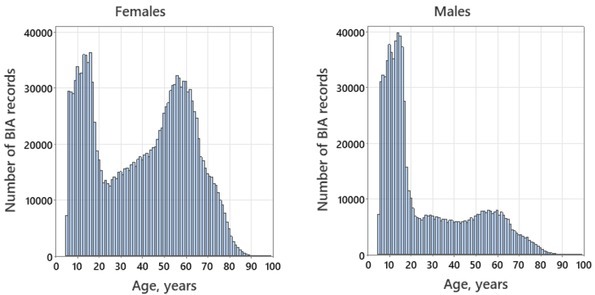
Age structure of the measured persons: left – females (n=1,607,906), right – males (n=822,071).

School-age children were examined in HCs most often, with the total number of boys slightly higher than that of girls (figure 5). After the school age, the number of examinations per year of age decreased sharply, and the rate of BIA measurements was dominated by females. The peak rate of the measurements among adults fell on a subgroup of women aged 50-65 years.

**Fig.6:**
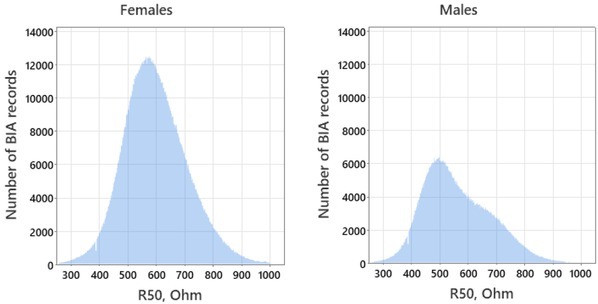
Histograms of the resistances R50: left – females (n=1,607,906), right – males (n=822,071).

Histograms of the resistance values at frequency 50 kHz are shown in figure 6. For the subgroup of males, the distribution was markedly different from normal (figure 6, right) due to a higher proportion of school-age children and, hence, of the measurement records with the bigger R50 values. Meanwhile, the median value of the resistance for a subgroup of females (590.4 ohm) was higher than that of males (544.7 ohm).

**Fig.7:**
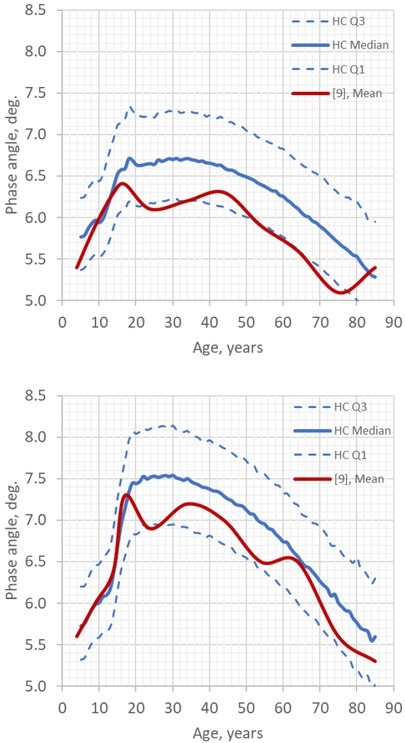
HCs’ reference data on the phase angle at 50 kHz according to age: the medians and interquartile range (blue lines). Upper panel – females (n=1,607,906), lower panel – males (n=822,071). The red lines show estimated mean values according to meta-analysis of pooled data from 46 studies [9].

Figure 7 shows HCs’ data on age-related changes of the phase angle values in males and females. In adults, these values were generally higher, and in children were similar to those obtained in a meta-analysis of pooled data from different countries involving more than 250,000 subjects [9].

## Discussion

The importance of BIA data lies in their availability and abundance for many countries. In particular, such data may allow us to obtain a holistic picture of the distributions of bioimpedance parameters, body composition estimates and associated health risks at the population level. This work presents the structure of the updated database of BIA measurements in Russian HCs.

Many HCs reported routine use of BIA as a part of comprehensive examination of every patient (in the presence of electrodes and serviceable instruments), while others reported a selective approach. In view of termination of the automated collection of HCs data at the federal level in 2014 and essentially manual data collection from HCs in this study (with a multiple written appeal to the governing bodies of HCs), the obtained BIA data were necessarily incomplete, and it took considerable amount of time to collect and preprocess the data.

Serious contamination of the BIA data by the outliers and fraud data was observed, which required the development and application of the efficient algorithms and software for their filtering [11]. In our view, falsification of the BIA data can mask low attendance of a HC (i.e., below the established plan) or lack of measurement electrodes. Interestingly, the quality of BIA data generated by various HCs obeyed the Pareto principle, so that the main body of the fraud data was generated by a relatively small number of HCs [11] which suggests the potential effectiveness of quality control of the HCs function based on the assessment of raw BIA data. Therefore, and also in view of the incompleteness of the data collected manually, it is important to restore automated collection of the HCs data at the federal level – for instance, through the connection of HCs to the Uniform State Health Information System (USHIS). It can be noted that the problem of generating fraudulent data is common to healthcare systems around the world and should not be neglected when analyzing massive aggregated biomedical data and making decisions [15].

The non-uniform attendance of HCs depending on age and sex can be noted (figure 5) that does not match the demographic structure of the Russian population, which is a result of free access to HCs for all citizens without any official regulations, especially in adults. For example, in figure 5, one can see a predominance of middle-aged and elderly women in the structure of HCs’ attendance compared to men of the same age which may reflect a more attentive attitude of women to their health. (Note that in Russia the average life expectancy among women steadily exceeds that of men by about 9-10 years [16].) The above discrepancy raises an important question of whether the obtained BIA data are representative. Our previous results suggest close similarity of the HCs’ data in school-age children to the general population data as exemplified by the analysis of the BIA records of a sample of Moscow children directly assessed in schools (n=1949): in this group, the mean standard deviation score for BMI relative to the Moscow HCs’ reference data described in [10] was as low as 0.06 and 0.09 in boys and girls, respectively [17]. A similar study needs to be conducted in adults.

One of the advantages of the HCs’ BIA database is the use of the same type of BIA instrument – ABC-01 'Medas'. Due to the lack of validated prediction formulae for the Russian population, appropriate foreign body composition formulae were utilized instead to generate relative body composition scales and local references [12,13]. Upon the development of the nationally representative formulae body composition estimates and local references will be updated using the obtained HCs’ BIA data.

Application of different BIA instruments and types of electrodes affects the result of BIA measurements [18,19]. Our data suggest an acceptable level of the reproducibility of the BIA measurement data obtained in HCs using different ECG electrodes and ABC-01 ‘Medas’ instruments which is similar to the inter-individual TEM reported in [20] for the Seca 515/514 instrument. Also, in the above-mentioned study at Medical Computer Systems company (Zelenograd, Russia) with the use of 8 types of electrodes, the mean differences in the phase angle between the other electrode types and the reference Bianostic AT (Data Input GmbH, Germany) electrode were all positive, between +0.1–0.2°, while other authors reported the negative differences at the level –0.1° for the 3M Red Dot 2330 and Ambu BlueSensor 2330 electrodes, and even –0.8° for the small skin contact area Ambu BlueSensor SU-00-C electrode [19] which emphasizes the importance of the proper choice of electrodes. So, the results of comparison of the phase angle data presented in figure 7 may be explained, at least in part, by the differences in electrode types (as mostly the lead-type instruments were included in the meta-analysis of pooled PA data from 46 studies [9]). Even the greater disagreement can be observed when using BIA instruments of different manufacturers, especially those utilizing distinct measurement approaches. The study at Medical Computer Systems company (Zelenograd, Russia) also included a comparison of the ABC-01 ‘Medas’ analyzer with the phase-sensitive Tanita MC-780A instrument (Tanita Corp., Japan), and the mean difference in PA values of +0.7° was obtained (lower values were observed with the Tanita instrument).

Federal subjects of Russia were presented in the BIA data relatively disproportionately (figure 2), whereas some Federal subjects were not involved at all. This is partly due to the use in HCs, along with the ABC-01 ‘Medas’ analyzer, of other bioimpedance instruments, manufactured mainly by the Diamant LLC company (Russia). These data, although also collected, need special attention as the Diamant instruments use untypical measurement frequencies (28 and 115 kHz) and the original scheme of electrode placement on arms and legs (along with the reusable electrodes of own production), so that the measured impedance values for the Diamant instrument are normally about half the values of those for the Medas instrument. Our preliminary results suggest comparability of the Medas and Diamant body composition data in adults at the group level after cross-calibration of measured impedances and application of the same assessment algorithm. Meanwhile, the proportion of explained variance using the conversion formula between the Medas and Diamant impedances in paired measurements (*r*^2^=0.90, unpublished data) was similar to that of the lead-type Medas vs standing-on Tanita BC-418MA (Tanita Corp., Japan) instrument (*r*^2^=0.91, [21]) and lower than previously reported in a classical work on the comparison of the lead-type RJL 101 (RJL Systems, USA) and Valhalla 1990B (Valhalla Scientific, USA) instruments involved in Fels Longitudinal Study and NHANES III, respectively, utilizing the same scheme of electrode placement (*r*^2^=0.99, [4]). Combined use of the Medas and Diamant data could be potentially important for studying the regional variability of bioelectric and body composition parameters in the population.

## Conclusion

The structure of the updated Russian HCs’ BIA database is presented. We believe that, after appropriate sampling, it can be utilized to study biological, geographical, social and other associations of the bioimpedance and body composition parameters, for generating updated national references, international comparisons and data standardization.
